# Supplementary material for: TrkB Agonist LM22A-4 Increases Oligodendroglial Populations During Myelin Repair in the Corpus Callosum
Source: Front Mol Neurosci. 2019 Aug 27;12:205. doi: 10.3389/fnmol.2019.00205 (PMC6718610; doi:10.3389/fnmol.2019.00205)
Supplement: Supplementary file 1 [file Data_Sheet_1.docx]

Supplementary Material

#
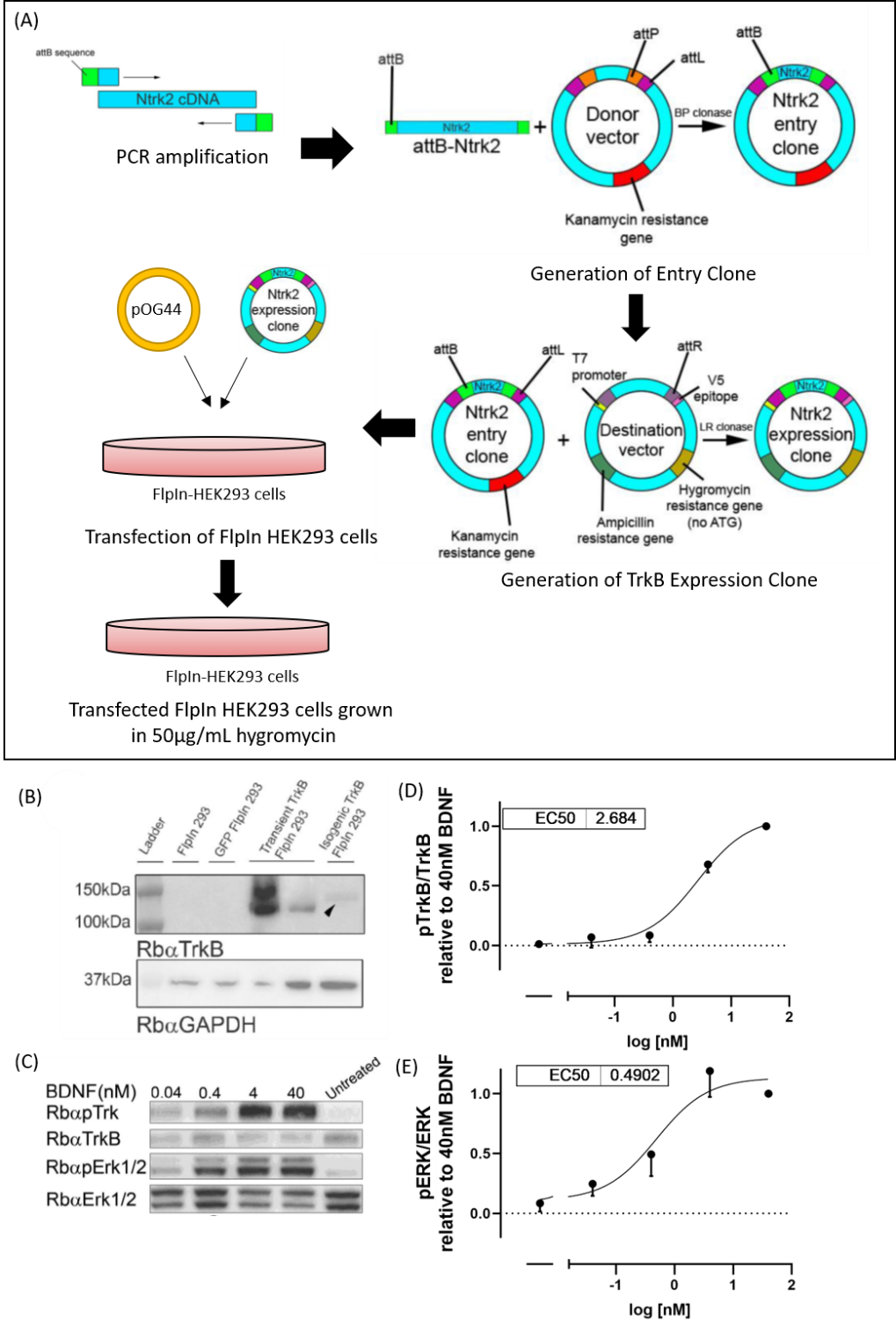
Supplementary Figures

**Supplementary Figure 1.** (A) Schematic of the generation of isogenic TrkB-293 cells using the Flp-In system. (B) Representative Western blot demonstrating that TrkB-293 cells stably express the mature glycosylated form of TrkB (arrowhead). Transient transfections occurred over 48 hours. (C) Representative Western blot showing that (D) TrkB and (E) ERK1/2 phosphorylation in TrkB-293 cells demonstrate a dose response to BDNF with an EC_50_ of 2.7nM and 0.5nM BDNF respectively.


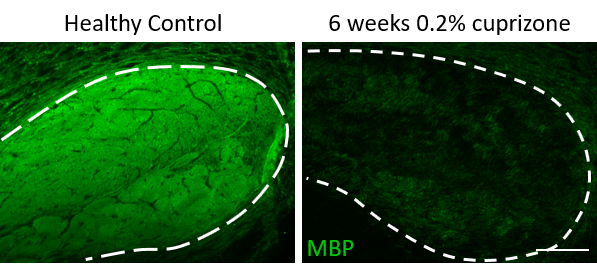


**Supplementary Figure 2.** Successful demyelination in the corpus callosum was confirmed with immunostaining for myelin basic protein (MBP). Min. *n*=2/cohort.
